# Polymer Micro/Nanofabrication and Manufacturing II

**DOI:** 10.3390/polym17182525

**Published:** 2025-09-18

**Authors:** Yi-Je Juang

**Affiliations:** Department of Chemical Engineering, National Cheng Kung University, Tainan 717005, Taiwan; yjjuang@mail.ncku.edu.tw

Polymer-based micro- and nanofabrication has surged forward in recent years, powered by demands for lightweight, adaptive, and multifunctional systems across applications ranging from energy harvesting [1,2], the construction of smart materials [3,4,5], tissue engineering [6,7], biomedical devices [8,9], and healthcare and diagnostics [10,11], to flexible electronics [12,13], wastewater purification [14], food safety inspection [15,16], and metasurfaces [17]. Polymers offer unparalleled versatility—they can be chemically engineered, shaped using diverse techniques, and endowed with responsiveness to external stimuli, making them central pillars in modern micro- and nanoscale manufacturing. Building upon these unique advantages, researchers have explored and established diverse patterning and structuring approaches. Although various polymer micro-/nanofabrication techniques based on fabricating structures using a mold, an energy beam, or mechanical machining/cutting have been reviewed [18], there is a crucial need to ensure high spatial precision and replication accuracy for industrial applications. While conventional techniques such as microinjection molding and micro-embossing still play imperative roles, the rapid evolution of additive manufacturing has introduced new paradigms for polymer structuring, and 3D/4D printing has established itself as an indispensable tool for polymer microfabrication [19,20]. This is attributed to its ability to fabricate complex geometries with high precision, enable rapid prototyping with minimal material waste, incorporate smart and stimulus-responsive functionalities to polymer structures, significantly shorten the design-to-production cycle, and support customization. This Special Issue, “Polymer Micro-/Nanofabrication and Manufacturing II”, features eleven high-quality papers that highlight innovative fabrication methods, surface engineering strategies, and potential applications spanning energy harvesting, flexible electronics, biomedical devices, and smart materials. Together, these contributions reflect the vibrant, interdisciplinary nature of the field and its continuing push towards precision, functionality, and scalability that echoes the focal points in this field. To address the issues regarding spatial precision and replication accuracy, studies on molding replicability and mold fabrication have been reported [21,22]. Zhu et al. used a microinjection mold with ultrasonic vibration to investigate the molding quality. It was found that better molding quality was achieved for transverse microstructures when ultrasonic vibration was applied perpendicularly to the polymer-melt filling direction [21], which is perhaps counterintuitive. Because the mold is the key to injection molding, Titu and Pop addressed the challenges of electrical erosion molding (EDM) for mold making, investigating and optimizing the processing conditions [22]. Parameters such as the discharge current and voltage, electrode material, and machining speed play crucial roles in determining productivity, electrode wear, and surface quality. The fabrication of well-defined nanogroove patterns on acrylic films was investigated [23]. Using a thermos oxide silicon mold with nanostructure patterns under optimized processing conditions, Raksiri et al. were able to generate nanogrooves with an average distance between the peaks of about 500 nm, average width of about 180 nm, and average height of about 105 nm without “slipping”. Again, the simplicity and directness of micro-embossing make it a valuable approach for the large-scale production of nanoscale patterns. Discussions related to precision and accuracy also extend to additive techniques. Kalilayeva et al. showed that different features and build orientations can affect the accuracy of the printed part differently in stereolithography [24]. For most simple geometries, deviations can be reduced using higher build angles. For complex features, 45° provides the most stable and reliable accuracy. Different from the previous study, Mukai et al. adjusted the scanning speed and type of macro-RAFT agents in stereolithography to achieve on-demand control of the phase-separated structures [25]. The authors also pointed out the usefulness of shrinkage compensation in multimaterial 3D printing for high-precision 3D printing. Different microstructures produced through stereolithography-based 3D printing were demonstrated for potential applications. Takenouchi et al. proposed a two-step approach for an oxidative polymerization and doping process where PTSA was used as the dopant to obtain a photocurable resin with a conductivity 100 times higher than that reported in the literature [26]. Then, flexible wiring was fabricated through 3D printing and demonstrated on a polyimide film. Ertugrul et al. constructed a strain sensor composed of channels produced from a conductive UV resin and flexible base from a non-conductive UV resin using a stereolithography (SLA)-based printer [27]. A close linear relationship between the strain sensor and the measured resistance was obtained. Ghelardini et al. created thermoresponsive hydrogel structures embedded with gold nanorods (GNRs) via 3D printing, where the shape change of the structures was enabled through photothermal heating [28]. We note that BSA-GNRs did not leach out of the structure during multiple cycles of shrinkage and reswelling. Huang et al. utilized a dual-pulse laser ablation technique to produce a polymethyl methacrylate (PMMA) mold where polydimethylsiloxane (PDMS) microneedles were created through casting the mold [29]. Compared with flat polydimethylsiloxane–aluminum triboelectric nanogenerators (PDMS-Al TENG), the dual-pulse PDMS-Al TENG increased the output power by 3.69 times, owing to an increase in the contact area between tribo-layers. This Special Issue includes insightful studies such as a study examining how heat transfer affects the performance of flexible electronics by Raczy’nski et al. [30] and a detailed characterization of collagen fibril diameter distribution for better understanding tissue mechanics by Smatov et al. [31]. These articles exemplify the advancements and broad applications of polymer micro-/nanofabrication technologies and manufacturing, which will undoubtedly expand the capabilities of micro-/nanofabricated polymers, fostering new solutions across healthcare, energy, electronics, and beyond. We anticipate that the insights presented in this Special Issue will continue to inspire and guide future research endeavors toward these exciting horizons.
